# Performance Degradation in Cross-Eye Jamming Due to Amplitude/Phase Instability between Jammer Antennas

**DOI:** 10.3390/s21155027

**Published:** 2021-07-24

**Authors:** Je-An Kim, Joon-Ho Lee

**Affiliations:** Department of Information and Communication Engineering, Sejong University, Seoul 05006, Korea; jean5348@sju.ac.kr

**Keywords:** cross-eye, jamming, amplitude ratio, phase difference, Taylor expansion

## Abstract

Cross-eye gain in cross-eye jamming systems is highly dependent on amplitude ratio and the phase difference between jammer antennas. It is well known that cross-eye jamming is most effective for the amplitude ratio of unity and phase difference of 180 degrees. It is assumed that the instabilities in the amplitude ratio and phase difference can be modeled as zero-mean Gaussian random variables. In this paper, we not only quantitatively analyze the effect of amplitude ratio instability and phase difference instability on performance degradation in terms of reduction in cross-eye gain but also proceed with analytical performance analysis based on the first order and second-order Taylor expansion.

## 1. Introduction

For monopulse tracking radars, the angular position of the target can be measured with one pulse. A monopulse radar measures the angular position of a target using a beam with four squint angles. The received signal is transmitted into four receiving antennas, each of which is transmitted into a sum channel and a difference channel, and monopulse radars can calculate azimuth and elevation information using the amplitude or phase of the channels [1]. In this way, because monopulse computes angles using only one transmission pulse, angle deception by the jammer system is difficult.

However, cross-eye jamming is one of the jamming techniques that can effectively deceive the angle estimation of these monopulses. Cross-eye jamming is an electronic attack (EA) technique that is used to induce an angular error in the radar being jammed by recreating the worst case glint angular error [2,3,4,5,6,7]. Cross-eye jamming is an angular deception technique that deceives monopulse radars as to the true position of their target by re-creating the worst angular error [8,9,10]. Angular deception is most often required in the final stages of an engagement where a platform is attempting to protect itself against radar-guided missiles. Cross-eye jamming is a method where two onboard antennas are used to modify the phase front in order to produce a false target near the real one. This process is called phase front distortion. Cross-eye jamming offers a general technique for countering monopulse radar tracking, but great efforts are required to produce a signal that is strong enough to overpower the real echo [11,12].

A cross-eye jamming scheme can be operated by placing two jamming antennas that generate jamming signals at a distance of *L*. A monopulse tracking radar also estimates the measurement angle using a difference pattern and sum pattern for signals generated by jamming antennas. This measured angle by the jamming antenna will cause it to deceive the angle of the true target. The ratio for the sum pattern of the jamming signal consists of the amplitude ratio and phase difference of the two signals, which can be used to obtain cross-eye gain. Thus, cross-eye’s jamming performance depends on amplitude ratio and phase difference.Cross-eye jamming is most effective with amplitude ratio of unity and phase difference of π. Mechanical defects can result in zero mean random Gaussian variables in the amplitude and phase set by cross-eye. As a result, cross-eye performance can be degraded, and the performance can be calculated according to the error.

In this paper, simulation MSD and analytic MSD are compared. Different standard deviations are applied to the amplitude ratio and phase difference to calculate cross-eye gain. This cross-eye gain can be approximated by using the first-order Taylor expansion and second-order Taylor expansion. Simulation MSD of these cross-eye gains can be obtained by the Monte Carlo simulation method. Analytic expressions of MSD are given by explicit expressions in terms of the variances of *a* and ϕ.

Many previous studies focused on how the cross-eye gain can be maximized to make the angle estimation error as large as possible. Our contribution in this manuscript does not concern how *a* and ϕ can be controlled to maximize the cross-eye gain.

Our contribution in this paper lies in a reduction in computational cost in getting the MSD of cross-eye gain by adopting an analytic approach, rather than the Monte Carlo simulation-based MSD under measurement uncertainty due to additive Gaussian noise. That is, the scheme described how analytic MSD can be obtained with much less computational complexity than the Monte Carlo simulation-based MSD.

Note that the proposed scheme in this paper is not a new cross-eye jamming algorithm with greater cross-eye gain than previous existing cross-eye jamming algorithms. The proposed scheme is on how the MSD of previously existing cross-eye jamming algorithms can be obtained analytically with much less computational complexity than the Monte Carlo simulation-based MSD.

To quantify the improvement in the computational cost, computational complexity in execution time is illustrated both for analytically derived MSD and for the Monte Carlo simulation-based MSD. Note that the computational complexity is independent of the standard deviation.

**Figure 1:**
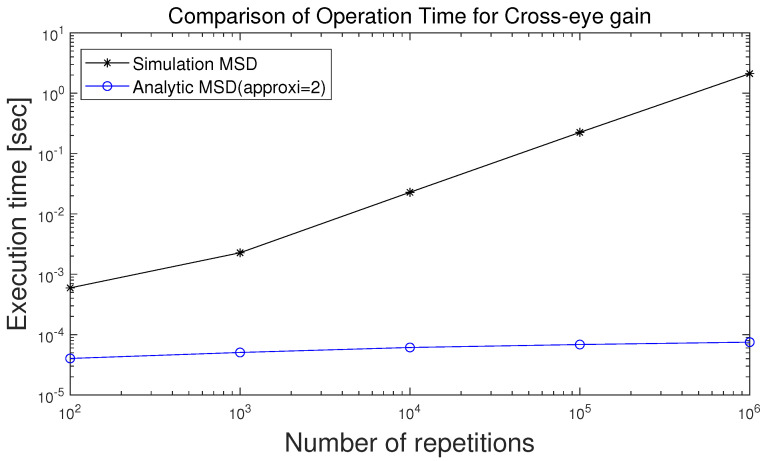
Execution time of MSD of cross-eye gain.

With regard to obtaining the Monte Carlo simulation-based MSD, the computational complexity is nearly proportional to the number of repetitions, and the results for the number of repetitions of 10, 100, 1000, 10,000 and 100,000 are shown. It is clearly shown in Figure 1 that the computational complexity for analytically derived MSD is much less than that for the Monte Carlo simulation-based MSD. Note that the execution time for analytically derived MSD is independent of the number of repetitions, which is why the execution time of the analytically derived MSD is flat with respect to the number of repetitions.

## 2. Cross-Eye Jamming Technique

In this section, it is shown how cross-eye gain can be expressed in terms of *a* and ϕ [1,8,13,14]. Note that the derivation in this section has been illustrated in many references [1,8,13,14], and it is not our contribution. The novelty in this paper lies in the derivations in Section 3 and Section 4. Although the derivation in Section 2 can also be found in [1,8,13,14], the derivations in Section 3 and Section 4 can not be found in [1,8,13,14].

**Figure 2:**
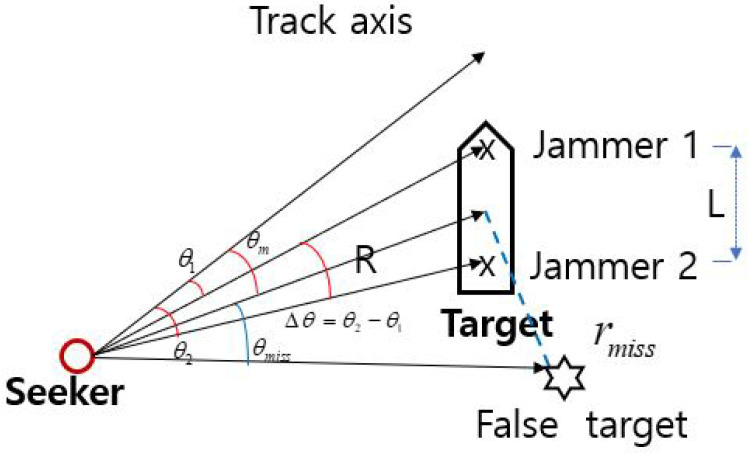
Cross-eye technique.

Figure 2 illustrates cross-eye’s scheme to place a jamming signal source that is *L* away from the target, deceiving the angle and distance information of the target in the monopulse radar. The indicated tracking angles $\theta_{1},\theta_{2}$ of the monopulse radar for the jamming signal produced by the two jamming antennas can be expressed as

(1) $$\begin{array}{c} \Delta_{1}=k_{m}\theta_{1}\Sigma_{1}\\ \Delta_{2}=k_{m}\theta_{2}\Sigma_{2}, \end{array}$$

where $k_{m}$ is the scale factor, and Δ and Σ are signals received in the sum and difference channels, respectively. The estimation angle of the monopulse algorithm can be written as

(2) $$\theta_{i}=\frac{1}{k_{m}}\frac{\Delta }{\Sigma }=\frac{\Delta_{1}+\Delta_{2}}{\Sigma_{1}+\Sigma_{2}}=\frac{\theta_{1}\Sigma_{1}+\theta_{2}\Sigma_{2}}{\Sigma_{1}+\Sigma_{2}}.$$

The ratio of the two sum channels is given in the form of a complex number, such as $\frac{\Sigma_{2}}{\Sigma_{1}}=ae^{j\phi }$. The amplitude and phase of the second jamming source relative to the first jamming source are *a* and ϕ, respectively. From (1) and (2), the indicated angle is given by

(3) $$\theta_{i}=\frac{\theta_{1}+ae^{j\phi }\theta_{2}}{1+ae^{j\phi }}.$$

Multiplying the right side of (3) by $\frac{\left(1+ae^{-j\phi }\right)}{\left(1+ae^{-j\phi }\right)}\;$ and applying the Euler formula, the indicated angle can be written as

(4) $$\theta_{i}=\theta_{m}-\frac{\Delta \theta }{2}\frac{1-2ja\sin\phi -a^{2}}{1+2a\cos\phi +a^{2}}.$$

The real part of (4) is the tracking angle that appears on the monopulse tracking radar due to jamming signals. This angle can be expressed as

(5) Re(θi)=θm−Δθ21−a21+2acosϕ+a2,

where $\theta_{m}$ is the angle from boresight to the point between the two scatterers, and Δθ is half the angular separation of two jammer antennas. The miss angle from the jammer is given as

(6) $$\theta_{\mathrm{miss}}=\frac{\Delta \theta }{2}\frac{1-a^{2}}{1+2a\cos\phi +a^{2}}.$$

Using the miss distance, $r_{\mathrm{miss}}$ is given by

(7) $$r_{\mathrm{miss}}=R\cdot \tan\left(\frac{\Delta \theta }{2}\frac{1-a^{2}}{1+2a\cos\phi +a^{2}}\right)$$

where *R* is the distance between the center point for two jammer antennas and target. Because a very small angle is applied to the tangent and $\frac{L\cos\psi }{R}$ is used instead of Δθ, $r_{\mathrm{miss}}$ can be approximated as

(8) $$r_{\mathrm{miss}}=\frac{L\cos\psi }{2}\frac{1-a^{2}}{1+2a\cos\phi +a^{2}},$$

where *L* is the distance between the two jammers, and ψ is the angle between the track axis and the line that bisects the jammers vertically. The cross-eye gain in expression in $\theta_{\mathrm{miss}}$ and $r_{\mathrm{miss}}$ is defined as

(9) $$GC\left(a,\phi \right)=\frac{1-a^{2}}{1+a^{2}+2a\cos\phi }.$$

Using (6), (8) and (9) can be compactly written as

(10) $$\theta_{\mathrm{miss}}=\frac{\Delta \theta }{2}GC$$

(11) $$r_{\mathrm{miss}}=\frac{L\cos\psi }{2}GC.$$

The cross-eye gain in (9) achieves the maximum value for *a* = 1 and $\phi =180^{\circ }$, which indicates that $\theta_{\mathrm{miss}}$ and $r_{\mathrm{miss}}$ also achieve the maximum for *a* = 1 and $\phi =180^{\circ }$.

## 3. Cross-Eye Gain by Approximation

As shown in (10) and (11), cross-eye jamming performance is highly dependent on the amplitude ratio between two jamming antennas and phase difference between two jamming antennas. Practically, the real amplitude ratio between two jamming antennas can be different from the nominal amplitude ratio between two jamming antennas. Similarly, the real phase difference between two jamming antennas can be different from the nominal phase difference between two jamming antennas. It is assumed that the error generated at this time follows a Gaussian distribution with an average of zero. If the amplitude ratio and phase difference initially set by cross-eye are $a_{0},\;\phi_{0}$ and real amplitude ratio and phase difference with errors are $a_{\mathrm{real}},\phi_{\mathrm{real}}$ each cross-eye gain can be calculated by entering each amplitude ratio and phase difference in (8).

(12) $$GC_{\mathrm{nominal}}\left(a_{0},\phi_{0}\right)=\frac{1-a_{0}^{2}}{1+a_{0}^{2}+2a_{0}\cos\phi_{0}}$$

(13) $$GC_{\mathrm{real}}\left(a_{\mathrm{real}},\phi_{\mathrm{real}}\right)=\frac{1-a_{\mathrm{real}}^{2}}{1+a_{\mathrm{real}}^{2}+2a_{\mathrm{real}}\cos\phi_{\mathrm{real}}}$$

Let the mean square difference (under MSD) denote the expectation of the square of the difference between the nominal gain and the real gain

(14) $$MSD=E\left[{\left(GC_{\mathrm{real}}-GC_{\mathrm{nominal}}\right)}^{2}\right],$$

where $GC_{\mathrm{nominal}}$ and $GC_{\mathrm{real}}$ are defined in (12) and (13), respectively. Note that MSD quantifies the perturbation of the cross-eye gain due to the fact that $a_{\mathrm{real}}$ and $\phi_{\mathrm{real}}$ are not exactly equal to $a_{0}$ and $\phi_{0}$, respectively. The approximate real cross-eye gain based on the first-order Taylor expansion are given by (15)

(15) GCreal(areal,ϕreal)(approxi=1)=GCnominal(a0,ϕ0)+∂GC(a,ϕ)∂aa=a0ϕ=ϕ0areal−a0+∂GC(a,ϕ)∂ϕa=a0ϕ=ϕ0ϕreal−ϕ0.

The coefficient of the first-order Taylor expansion can be expressed as

(16) ∂GC(a,ϕ)∂aa=a0ϕ=ϕ0=αa=−2cosϕ0a02+2a0+cosϕ01+2cosϕ0a0+a022∂GC(a,ϕ)∂ϕa=a0ϕ=ϕ0=αϕ=−(2a0sinϕ0(a02−1))1+2cosϕ0a0+a022.

Using (16), the first-order Taylor series can be simplified to

(17) $$GC_{\mathrm{real}}^{\left(\mathrm{approxi}=1\right)}=GC_{\mathrm{nominal}}+\alpha_{a}\left(a_{\mathrm{real}}-a_{0}\right)+\alpha_{\phi }\left(\phi -\phi_{0}\right).$$

Similarly, based on the second-order Taylor series, the cross-eye gain for the real amplitude ratio and real phase difference can be approximated as

(18) GCreal(areal,ϕreal)(approxi=2)=GCnominal(a0,ϕ0)+∂GC(a,ϕ)∂aa=a0ϕ=ϕ0areal−a0+∂GC(a,ϕ)∂ϕa=a0ϕ=ϕ0ϕreal−ϕ0+12∂2GC(a,ϕ)∂a2a=a0ϕ=ϕ0areal−a02+2∂GC(a,ϕ)∂a∂GC(a,ϕ)∂ϕa=a0ϕ=ϕ0areal−a0ϕreal−ϕ0+∂2GC(a,ϕ)∂ϕ2a=a0ϕ=ϕ0ϕreal−ϕ02.

The coefficient of the second-order Taylor expansion can be expressed as $\beta_{a}$, and $\beta_{a\phi }$ and $\beta_{\phi }$ are coefficients of the second-order Taylor expansion:

(19) ∂2GC(a,ϕ)∂a2a=a0ϕ=ϕ0=βa=8cosϕ02+12a0cosϕ0+12a02+4a03cosϕ0−41+2cosϕ0a0+a023∂GC(a,ϕ)∂a∂GC(a,ϕ)∂ϕa=a0ϕ=ϕ0=βaϕ=−2sinϕ0−a04+2cosϕ0a03+6a02+2cosϕ0a0−11+2cosϕ0a0+a023∂2GC(a,ϕ)∂ϕ2a=a0ϕ=ϕ0=βϕ=−2a0a02−14a0+cosϕ0−2a0cosϕ02+a0cosϕ01+2cosϕ0a0+a023.

Using (19), the first-order Taylor series can be simplified to

(20) $$GC_{\mathrm{real}}^{\left(\mathrm{approxi}=2\right)}=GC_{\mathrm{nominal}}+\left(a_{a}\left(a_{\mathrm{real}}-a_{0}\right)+\alpha_{\phi }\left(\phi_{\mathrm{real}}-\phi_{0}\right)\right)\;+\left[\begin{array}{c} \beta_{a}{\left(a_{\mathrm{real}}-a_{0}\right)}^{2}+\beta_{a\phi }\left(a_{\mathrm{real}}-a_{0}\right)\left(\phi_{\mathrm{real}}-\phi_{0}\right)\\ +\beta_{\phi }{\left(\phi_{\mathrm{real}}-\phi_{0}\right)}^{2} \end{array}\right].$$

## 4. Analytic Expression MSD of the Cross-Eye Gain

In this paper, we propose a scheme to quantify how much reduction in cross-eye gain occurs due to the perturbations in the amplitude ratio and the phase difference. The amount of reduction in cross-eye gain can be obtained from the Monte Carlo simulation, which can be computationally intensive, especially for a large number of repetitions in the Monte Carlo simulation. Therefore, a computationally efficient approach to quantify the reduction in cross-eye gain is proposed. The difference between the real cross-eye gain and the nominal cross-eye gain is written as

(21) $$GC_{\mathrm{real}}-GC_{\mathrm{nominal}}=\frac{1-a_{\mathrm{real}}^{2}}{1+a_{\mathrm{real}}^{2}+2a_{\mathrm{real}}\cos\phi_{\mathrm{real}}}-\frac{1-a_{0}^{2}}{1+a_{0}^{2}+2a_{0}\cos\phi_{0}}.$$

In this section, we derive explicit expressions of the MSD. MSD through simulation is as follows

(22) $$E\left[{\left(GC_{\mathrm{real}}-GC_{\mathrm{nominal}}\right)}^{2}\right]=E\left[{\left(\frac{1-a_{\mathrm{real}}^{2}}{1+a_{\mathrm{real}}^{2}+2a_{\mathrm{real}}\cos\phi_{\mathrm{real}}}-\frac{1-a_{0}^{2}}{1+a_{0}^{2}+2a_{0}\cos\phi_{0}}\right)}^{2}\right].$$

The empirical MSD is given by

(23) $$\mathrm{MSD}=\frac{1}{N}\overset{N}{\underset{i=1}{\Sigma }}\left({\left(GC_{\mathrm{real}}{\left(a_{\mathrm{real}},\phi_{\mathrm{real}}\right)}_{i}-GC_{\mathrm{nominal}}\left(a_{0},\phi_{0}\right)\right)}^{2}\right)=\mathrm{Simulation}\;E\left[{\left(GC_{\mathrm{real}}{\left(a_{\mathrm{real}},\phi_{\mathrm{real}}\right)}_{i}-GC_{\mathrm{nominal}}\left(a_{0},\phi_{0}\right)\right)}^{2}\right],$$

where the lower-script (*i*) denotes the real cross-eye gain associated with the *i*-th repetition out of N repetitions. The first term of (17) and (20) is $GC_{\mathrm{nominal}}$ in (12). Therefore, when we calculate MSD for the first-order Taylor expansion and the second-order Taylor expansion, (12) is subtracted and only the difference part is left. The MSD of first order expansion is given as

(24) $$E\left[{\left(GC_{\mathrm{real}}^{\left(\mathrm{approxi}=1\right)}-GC_{\mathrm{nominal}}\right)}^{2}\right]=E\left[{\left(\alpha_{a}\left(a-a_{0}\right)+\alpha_{\phi }\left(\phi -\phi_{0}\right)\right)}^{2}\right]$$

Explicit expression of the MSD from the first-order Taylor expansion in terms of the variances of *a* and ϕ is given in (A2) in the Appendix A. The MSD of the second-order Taylor expansion is given as

(25) $$E\left[{\left(GC_{\mathrm{real}}^{\left(\mathrm{approxi}=2\right)}-GC_{\mathrm{nominal}}\right)}^{2}\right]=E\left[{\left(\begin{array}{c} \left(a_{a}\left(a-a_{0}\right)+\alpha_{\phi }\left(\phi -\phi_{0}\right)\right)+\\ \left[\beta_{a}{\left(a-a_{0}\right)}^{2}+\beta_{a\phi }\left(a-a_{0}\right)\left(\phi -\phi_{0}\right)+\beta_{\phi }{\left(\phi -\phi_{0}\right)}^{2}\right] \end{array}\right)}^{2}\right].$$

(A3) in the Appendix A is an explicit expression of the MSD from the second-order Taylor expansion in terms of the variances of *a* and ϕ.

## 5. Numerical Results of Cross-Eye Gain

This section shows the performance analysis of simulation MSD and analytic MSD. For the simulation, to give various cases, several standard deviation values of $a_{\mathrm{real}}$ and $\phi_{\mathrm{real}}$ are set. The simulation is performed in a nested loop, fixing one value and changing the other. It has various standard deviation values, and a performance analysis is performed for perturbed amplitude and phase. The results based on the Monte Carlo simulation is compared with those based on the analytical approach both for the first-order Taylor expansion and the second-order Taylor expansion.

**Figure 3:**
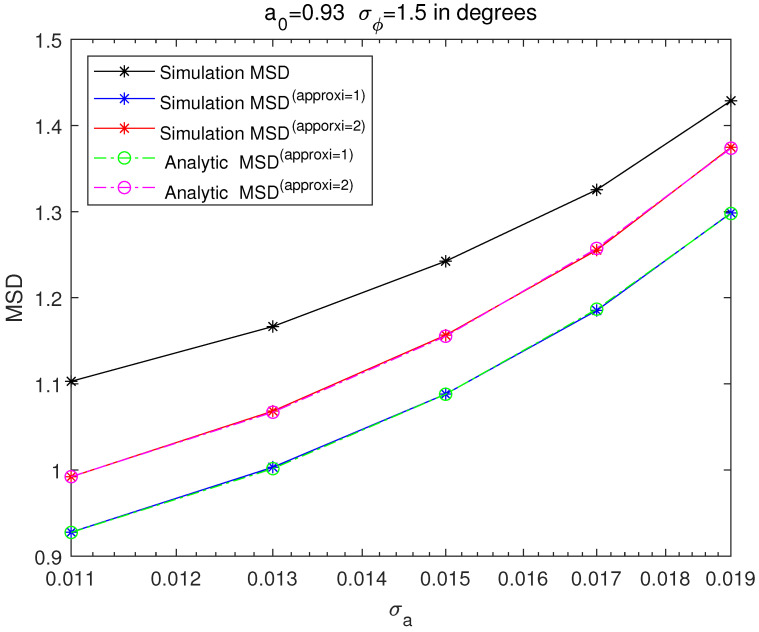
MSD of cross-eye gain, $\sigma_{\phi }$ fixed to $1.5^{\circ }$ degrees.

**Figure 4:**
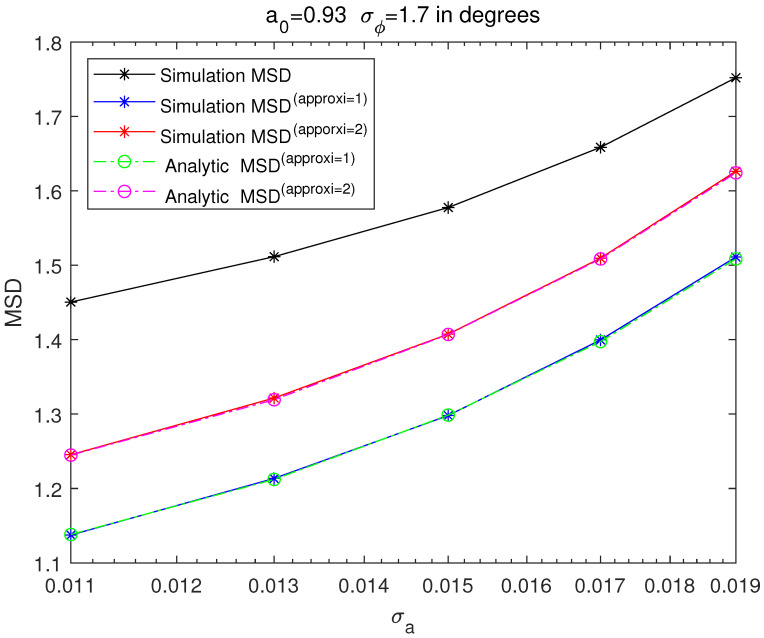
MSD of cross-eye gain, $\sigma_{\phi }$ fixed to $1.7^{\circ }$ degrees.

**Figure 5:**
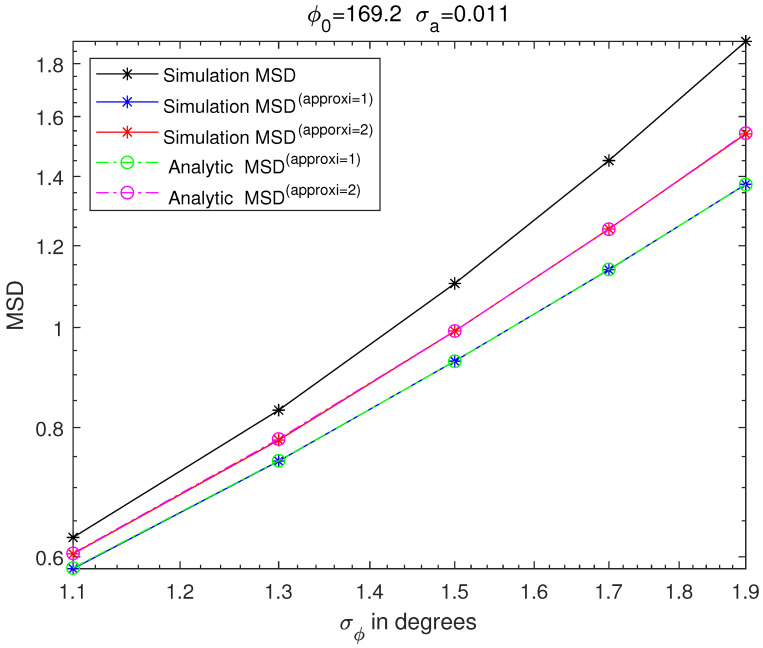
MSD of cross-eye gain, $\sigma_{a}$ fixed to 0.0011.

**Figure 6:**
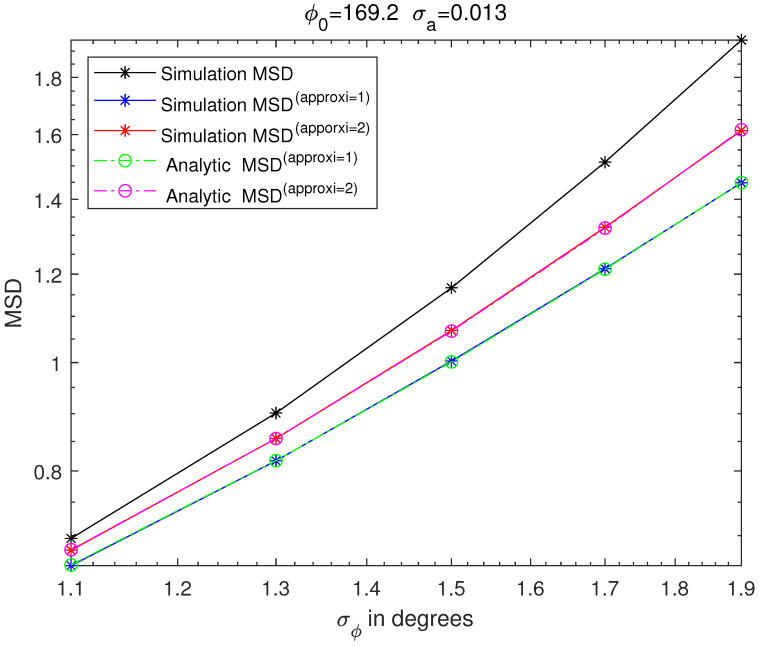
MSD of cross-eye gain, $\sigma_{a}$ fixed to 0.0013.

Figure 3, Figure 4, Figure 5 and Figure 6 are MSD graphs for the standard deviation of each amplitude ration and phase difference. The values of the standard deviation are determined as the result in Figure 3, Figure 4, Figure 5 and Figure 6 where ‘SimulationMSD’, ‘$\mathrm{Simulation}\;{\mathrm{MSD}}^{\left(\mathrm{approxi}=1\right)}$’, ‘$\mathrm{Simulation}\;{\mathrm{MSD}}^{\left(\mathrm{approxi}=2\right)}$’, ‘$\mathrm{Analytic}\;{\mathrm{MSD}}^{\left(\mathrm{approxi}=1\right)}$’ and ‘$\mathrm{Analytic}\;{\mathrm{MSD}}^{\left(\mathrm{approxi}=2\right)}$’ are obtained from (23)–(25), (A2) and (A3). Figure 3 and Figure 4 are the resulting graphs when the standard deviation of $\phi_{\mathrm{real}}$ is fixed and the standard deviation of $a_{\mathrm{real}}$ is changed. Figure 5 and Figure 6 are the resulting graphs when the standard deviation of $a_{\mathrm{real}}$ is fixed and the standard deviation of $\phi_{\mathrm{real}}$ is changed. Simulations are proceeded using (23), and simulations of the first-order Taylor approximations and the second-order Taylor approximations can be obtained by applying (17) and (20) to (23), respectively.

Because the first-order Taylor approximation is used to get $\mathrm{Simulation}\;{\mathrm{MSD}}^{\left(\mathrm{approxi}=1\right)}$ from ‘SimulationMSD’, ‘SimulationMSD’ is not equal to ‘$\mathrm{Simulation}\;{\mathrm{MSD}}^{\left(\mathrm{approxi}=1\right)}$’. Likewise, since the second-order Taylor approximation is used to get ‘$\mathrm{Simulation}\;{\mathrm{MSD}}^{\left(\mathrm{approxi}=2\right)}$’ from ‘SimulationMSD’, ‘SimulationMSD’ is not equal to ‘$\mathrm{Simulation}\;{\mathrm{MSD}}^{\left(\mathrm{approxi}=2\right)}$’. ‘$\mathrm{Simulation}\;{\mathrm{MSD}}^{\left(\mathrm{approxi}=1\right)}$’ and ‘$\mathrm{Analytic}\;{\mathrm{MSD}}^{\left(\mathrm{approxi}=1\right)}$’ show excellent agreements, which validates (17). Similarly, ‘$\mathrm{Simulation}\;{\mathrm{MSD}}^{\left(\mathrm{approxi}=2\right)}$’ and ‘$\mathrm{Analytic}\;{\mathrm{MSD}}^{\left(\mathrm{approxi}=2\right)}$’ show excellent agreement, which validates (20). Analytic MSD can be obtained with much less computational complexity than Monte Carlo simulation-based MSD. Not only that, it is clear that the results with the superscript ‘approxi=2’ are closer than the results with the superscript ‘approxi=1’ to the ‘SimulationMSD’. It means that the second-order Taylor approximation is more accurate than the first-order Taylor approximation.

Figure 1 shows the execution time of Monte Carlo simulation-based MSD of cross-eye gain and execution time of analytically derived MSD, respectively. In obtaining the Monte Carlo simulation-based MSD, the execution time is proportional to the number of repetitions. However, because analytically derived MSD is independent of the number of repetitions, the execution time of the analytically derived MSD is flat regardless of the number of repetitions. This can be seen clearly in Figure 1. It is clearly shown in Figure 1 that the computational complexity for analytically derived MSD is much less than that for the Monte Carlo simulation-based MSD with the number of repetitions of 1,000,000.

## 6. Conclusions

The cross-eye jamming technique can deceive angle tracking of a monopulse radar by transmitting jamming signals from two jamming antennas. Transmitting the jamming signal’s amplitude and phase affects the jamming performance. This can be confirmed in (6) and (8). However, due to mechanical defects, there is a difference between nominal cross-eye gain and real cross-eye gain. As a result, the change of the cross-eye gain, which is highly affected by amplitude ratio and phase difference, results in performance degradation. Therefore, a study was conducted on calculating MSD to know the cross-eye jamming performance in various different situations. Cross-eye jamming performance can be predicted by using the first-order Taylor series and the second-order Taylor series. The Monte Carlo simulation-based performance analysis is computationally intensive. To obviate this problem, a computationally efficient analytic approach to quantify cross-eye jamming performance has been proposed in this paper. As illustrated in Figure 1, the computational burden of the analytic approach is much smaller than that of the Monte Carlo simulation-based approach, especially for a large number of repetitions. Note that, for the Monte Carlo simulation-based MSD to be reliable, the number of repetitions should be large enough. Furthermore, it is also illustrated in the numerical results that the second-order Taylor series-based approach results in an accuracy improvement in comparison with the first-order Taylor series-based approach. The performance analysis proposed in this paper can be adopted to quantitatively describe the amount of degradation in cross-eye gain for the cross-eye jamming system due to some perturbations in the amplitude ratio and phase difference.

The usefulness of the derived expression is that the MSDs of the cross-eye jamming algorithm can be available from the derived expression without actually performing the computationally intensive Monte Carlo simulation, which is illustrated in the numerical results. By using the derived expression, we can get a quantitative measure of the difference between the nominal gain and the real gain without actually performing the computationally intensive Monte Carlo simulation.

To quantify the computational reduction in obtaining the MSD analytically in comparison with the Monte Carlo simulation-based MSD, the execution time with respect to the number of repetitions in the Monte Carlo simulation is illustrated in Figure 1, where the reduction in computational complexity associated with the analytically derived MSD in comparison with the Monte Carlo simulation-based MSD is clearly shown.

The proposed scheme can be used for the performance analysis in predicting how much degradation in cross-eye gain occurs due to the difference between the nominal values of *a* and ϕ and the real values of *a* and ϕ without resorting to a computationally intensive Monte Carlo simulation. Making Monte Carlo simulations for different values of the various parameters is very intensive computationally, and the analytic performance analysis proposed in this paper can be employed to quantitatively predict how much degradation in cross-eye gain results when the perturbations in *a* and ϕ are modeled as Gaussian random variables.

We rigorously derive how the MSD of the cross-eye jamming algorithm can be expressed in terms of nominal values of *a* and ϕ, the real values of *a* and ϕ and the statistics of the real values of *a* and ϕ in (A2) and (A3) for the first-order Taylor expansion and the second-order Taylor expansion, respectively.

Although, for convenience, the real values of *a* and ϕ are assumed to be Gaussian distributed, the derivation in the Appendices can be easily extended to the case where *a* and ϕ can be modeled as any other random variable as long as the moments of the random variable are analytically available.

## Appendix A. The Explicit Expression of the MSDs Based on the Taylor Second-Order Approximation

In this appendix, the MSD of the first-order and second-order Taylor approximation-based cross-eye gain in equation is expanded to obtain the Analytic MSD. It is assumed that the difference between the real amplitude ratio and nominal amplitude ratio are Gaussian random variables. Likewise, it is assumed that the difference between the real phase and nominal phase are Gaussian random variables. $\delta_{a}$ and $\delta_{\phi }$, which are differences between real and nominal, are distributed normally with a mean of 0 and variance $\sigma_{a}$ and $\sigma_{\phi }$, respectively.

(A1) $$\begin{array}{c} \delta_{a}∼N\left(0,\sigma_{a}\right)\\ \delta_{\phi }∼N\left(0,\sigma_{\phi }\right) \end{array}$$

The explicit expressions of MSDs are expressed as (A2) and (A3).

(A2) $$\begin{array}{ll} E\left[{\left(GC_{\mathrm{real}}^{\left(\mathrm{approxi}=1\right)}-GC_{\mathrm{nominal}}\right)}^{2}\right] & =E\left[{\left(\alpha_{a}\delta_{a}+\alpha_{\phi }\delta_{\phi }\right)}^{2}\right]=E\left[\alpha_{a}^{2}\delta_{a}^{2}+2\left(\alpha_{a}\delta_{a}\right)\left(\alpha_{\phi }\delta_{\phi }\right)+\alpha_{\phi }^{2}\delta_{\phi }^{2}\right]\;\\ & =\alpha_{a}^{2}E\left(\delta_{a}^{2}\right)+2\alpha_{a}\alpha_{\phi }E\left(\delta_{a}\delta_{\phi }\right)+\alpha_{\phi }^{2}E\left(\delta_{\phi }^{2}\right)\\ & =\alpha_{a}^{2}\sigma_{a}^{2}+\alpha_{\phi }^{2}\sigma_{\phi }^{2} \end{array}$$

(A3) $$\begin{array}{c} E\left[{\left(GC_{\mathrm{real}}^{\left(\mathrm{approxi}=2\right)}-GC_{\mathrm{nominal}}\right)}^{2}\right]\\ =E\left[{\left(\left(\alpha_{a}\delta_{a}+\alpha_{\phi }\delta_{\phi }\right)+\frac{1}{2}\left[\beta_{a}\delta_{a}^{2}+2\beta_{a\phi }\delta_{a}\delta_{\phi }+\beta_{\phi }\delta_{\phi }^{2}\right]\right)}^{2}\right]\\ =E\left[\begin{array}{c} \alpha_{a}^{2}\delta_{a}^{2}+\left(\alpha_{a}\alpha_{\phi }\right)\left(\delta_{a}\delta_{\phi }\right)+\frac{1}{2}\left(\alpha_{a}\beta_{a}\right)\delta_{a}^{3}+\left(\alpha_{a}\beta_{a\phi }\right)\left(\delta_{a}^{2}\delta_{\phi }\right)+\frac{1}{2}\left(\alpha_{a}\beta_{\phi }\right)\left(\delta_{a}\delta_{\phi }^{2}\right)+\\ \left(\alpha_{a}\alpha_{\phi }\right)\left(\delta_{a}\delta_{\phi }\right)+\alpha_{\phi }^{2}\delta_{\phi }^{2}+\frac{1}{2}\left(\alpha_{\phi }\beta_{a}\right)\left(\delta_{a}^{2}\delta_{\phi }\right)+\left(\alpha_{\phi }\beta_{a\phi }\right)\left(\delta_{a}\delta_{\phi }^{2}\right)+\frac{1}{2}\left(\alpha_{\phi }\beta_{\phi }\right)\delta_{\phi }^{3}+\\ \frac{1}{2}\left(\alpha_{a}\beta_{a}\right)\delta_{a}^{3}+\frac{1}{2}\left(\alpha_{\phi }\beta_{a}\right)\left(\delta_{a}^{2}\delta_{\phi }\right)+\frac{1}{4}\beta_{a}^{2}\delta_{a}^{4}+\frac{1}{2}\left(\beta_{a}\beta_{a\phi }\right)\left(\delta_{a}^{3}\delta_{\phi }\right)+\frac{1}{4}\left(\beta_{a}\beta_{\phi }\right)\left(\delta_{a}^{2}\delta_{\phi }^{2}\right)+\\ \left(\alpha_{a}\beta_{a\phi }\right)\left(\delta_{a}^{2}\delta_{\phi }\right)+\left(\alpha_{\phi }\beta_{a\phi }\right)\left(\delta_{a}\delta_{\phi }^{2}\right)+\frac{1}{2}\left(\beta_{a}\beta_{a\phi }\right)\left(\delta_{a}^{3}\delta_{\phi }\right)+\beta_{a\phi }^{2}\left(\delta_{a}^{2}\delta_{\phi }^{2}\right)+\frac{1}{2}\left(\beta_{a\phi }\beta_{\phi }\right)\left(\delta_{a}\delta_{\phi }^{3}\right)\\ \frac{1}{2}\left(\alpha_{a}\beta_{\phi }\right)\left(\delta_{a}\delta_{\phi }\right)+\frac{1}{2}\left(\alpha_{\phi }\beta_{\phi }\right)\delta_{\phi }^{3}+\frac{1}{4}\left(\beta_{a}\beta_{\phi }\right)\left(\delta_{a}^{2}\delta_{\phi }^{2}\right)+\frac{1}{2}\left(\beta_{a\phi }\beta_{\phi }\right)\left(\delta_{a}\delta_{\phi }^{3}\right)+\frac{1}{4}\beta_{\phi }^{2}\delta_{\phi }^{4} \end{array}\right]\\ =E\left[\begin{array}{c} \alpha_{a}^{2}\delta_{a}^{2}+\alpha_{\phi }^{2}\delta_{\phi }^{2}+\frac{1}{4}\beta_{a}^{2}\delta_{a}^{4}+\frac{1}{4}\left(\beta_{a}\beta_{\phi }\right)\left(\delta_{a}^{2}\delta_{\phi }^{2}\right)+\beta_{a\phi }^{2}\left(\delta_{a}^{2}\delta_{\phi }^{2}\right)+\frac{1}{4}\left(\beta_{a}\beta_{\phi }\right)\left(\delta_{a}^{2}\delta_{\phi }^{2}\right)\\ +\frac{1}{4}\beta_{\phi }^{2}\delta_{\phi }^{4} \end{array}\right]\\ =\left(\alpha_{a}^{2}\sigma_{a}^{2}+\alpha_{\phi }^{2}\sigma_{\phi }^{2}\right)+\left[\frac{3}{4}\beta_{a}^{2}\sigma_{a}^{4}+\frac{1}{2}\left(\beta_{a}\beta_{\phi }\right)\left(\sigma_{a}^{2}\sigma_{\phi }^{2}\right)+\beta_{a\phi }^{2}\left(\sigma_{a}^{2}\sigma_{\phi }^{2}\right)+\frac{3}{4}\beta_{\phi }^{2}\sigma_{\phi }^{4}\right] \end{array}$$
